# Autopsies at Central Park Menagerie

**Published:** 1887-07

**Authors:** 


### CASE DEPARTMENT.

### AUTOPSIES AT CENTRAL PARK MENAGERIE.

Chacma Baboon—*Cynocephalus porcarius*, *Hab.* West Africa. General emaciation.

*Thorax.—Left.* No adhesions. *Lung*-congested and rather solid. A few pin-point-sized tubercles in *pleura.* Abundant firm adhesions over lower part of lung. Well marked cheesy degeneration; one mass is filbert sized. *Right lung.*—Same. *Pleura.*—Costal, diaphragmatic and visceral; is infiltrated with pin-head sized greyish nodules. *Abdomen.—Omentum* contains moderate amount of fat; no adhesions. *Liver.*—Capsule shows a moderate number of pin-point to pin-head-sized tubercles. Organ fatty and congested. *Kidneys.*—Congested. *Mediastinal and Abdominal Lymphatic Glands.*—Swollen and congested. Many contain cheesy degenerated centers. *Intestines.*—Moderately congested. Large intestine contains a number of pea-sized, blackish-brown tumors, cheesy at the centres. No ulcerations. *Diagnosis.*—General tubercular infection. Death from exhaustion.

Paradoxure—*Paradoxurus bondar*, *Hab.* India. The interesting point is the intestinal obstruction which caused death by starvation. In the large intestine was an intussusception, half an inch in length, but without any peritonitis. It probably occurred during the last hours of life.

The small intestine, however, was stuffed with *hair-balls*, so as to completely occlude its lumen. The jejunum was chiefly involved, andwhile the stomach and duodenum contained a large amount of semi fluid food, the ileum and the large intestine contained only a little mucous. The substance of these hair-balls were so densely matted together that they could hardly be cut with the scissors. One was 2x1-2 inches in size, and another larger one was wedged into the intussuscepted portion of the large intestine.

In all five of these hair masses were found. All other organs were normal.

*Diagnosis.*—Intestinal obstruction by hair balls. *Cause of death.* —Inanition.

Ocelot—*Felis pardalis*, *Hab.* South America. Poor condition. Fur loose. Skin blood-stained over abdomen. *Lung.* Acute pneumonia the immediate cause of death. The entire pyloris end of the stomach was filled with a large irregular ball of hair, 2 1-2 by 1 1-2 inches in size. It almost entirely blocked the entrance to the duodenum. It is of interest in connection with the last case, and the two go to show the frequency of intestinal obstruction in these mammals from this habit of licking their coats.

Bear—*Ursus americanus*, *Hab.* North America. Dead thirty-six hours. Apparently sick for several days, showing signs of uneasiness. Fairly fat. *Thorax.—Pleura* moderately thickened. *Lungs.*—Base, left, acute congestion. *Heart.*—R. auriculo, ventricular valve nodular, thickened and covered with warty growths. *Endocardium* of L. heart, thickened; base of L. auriculo-ventricular valve adherent to wall of auricle. Closure imperfect. *Stomach.* —Full of watery fluid; no food. Contains five peach stones, one of which is impacted in the pylorus and fills it.

The entire mucous membrane of the whole stomach is intensely inflamed. For four inches back from the pylorus the stomach is enormously thickened, the increased tissue being chiefly of the middle coat. It is a grey, translucent material, moderately hard. It is disseminated through the whole area involved, but forms a large ring-shaped mass around the pylorus, especially on its posterior wall. The posterior part of the ring nearest the orifice has a projection or nipple, half an inch in height, tending to close the orifice. Between the nipple and the contracted pylorus lies the peach stone. *Intestines.*—Intensely inflamed in places. *Liver.*—Congested and fatty.

*Diagnosis.*—Colloid carcinoma of stomach. Ac. Gastritis. Intestinal obstruction by peach stones. *Cause of death.*—Exhaustion.

William S. Gottheil, M. D.,
New York City.

II.—Fracture of Radius in Brood-Mare.—*Amputation and Recovery.*—On April third I was called to the country to see a brood-mare with a fractured forearm, and found the following interesting case, which demonstrates the fallacy of the rapid unfavorable prognosis and slaughter which usually takes place when a valuable breeding animal breaks its leg.

April 3d, 11 p. m.

Gray mare, 15 1, 10 years old, within six weeks of foaling, having been covered in June last by a valuable horse. The mare had been turned into the barn-yard the morning before, and was found in a half hour with a fractured forearm. She was standing in rude slings, which pressed too much on the abdomen, and rendered her very uncomfortable. The off front leg was encased in a splint which had been admirably applied by the family physician. On removing the splint, I found a compound comminuated fracture of the lower end of the radius. The leg was considerably swollen and very painful. On attempting to examine the fracture the mare reared, dropped backward from the slings on to her sound side, from which it was impossible to raise her, as all but one attendant declined positively to see the animal suffer. I reapplied the splint, and early the following morning reported to the owner, that the only possible means of saving the foal would be amputation. April 4th, 8 A. M. mare on near side, nervous and suffering greatly ; has been sweating constantly through the night. Straining somewhat, but movements of fœteus can be felt. Hobbled hind legs and near (under) foreleg with ordinary barn rope; had head restrained by bridle and twitch. Made rapid circular operation in middle of forearm, cutting the skin and subcutaneous tissue in first sweep, the superficial layer muscles in second, and with the third, cut the the deeper muscles to the bone, sawed the bone with a saw borrowed from the farm kitchen, which was previously flamed to cleanse it. Was only obliged to ligate the anterior and posterior radial arteries, the remaining hæmorrhage answering promptly to cold water, rendered antiseptic by the addition of a small quantity of bichloride of mercury. United soft parts by two deep sutures leaving drainage at corners. Lifted mare to her feet and placed a loose narrow sling, under thorax. In an hour the mare had cooled from her sweating; was much less nervous, and ate a handful of oats, drank water and picked at a bundle of hay. A great deal of trembling continued in the near foreleg from the strain imposed upon it, as the animal declined to give herself much support from the hind legs. Left orders that the slings should be removed at night and the mare turned loose in her box stall.

During the following three weeks the animal was let down at night, and supported with slings in the day time, once or twice she was allowed to remain on her side for more than a day, and swelled a good deal in the mammæ and gentils.

April 24th, 10 P. M. Found wound almost healed, but muscles considerably contracted, allowing protrusion of bone which is, however, covered with a dense fibrous tissue. Find that animal has been constantly in slings during the day time, and that she depends on them, throwing the entire weight of the anterior part of the body into the canvas, instead of using her leg. Find also that she has given great trouble in having to be lifted to her feet each morning. Removed slings, in which mare was falling asleep. After several attempts she got down, showing, however, a great deal of caution for the amputated leg. In a few moments she was sound asleep.

April 25th. Animal on side bright; lifted head on our entrance into the stall, got herself ready to be lifted up, but refused to give any aid. Placed a bridle on her, struck her suddenly with a whip and she got up without aid. Walked her into barnyard and turned her loose. On May 1st she foaled without trouble, and is now at pasture with a healthy foal beside her. Before foaling, the mare used her hind legs but little for support, but since she no longer has to protect the abdomen, she has assumed more the gait of a foundered horse. The long stump of bone was obligatory on account of the awkward saw, which was the only one at hand; constant irrigation was not possible, but the animal received frequent bathing and excellent nursing.

In the above case the pregnancy proved a serious complication, but the animal fortunately had great courage. In future cases, I would advise but little use of slings, as the animal should learn to depend upon itself at once. In the case of a valuable breeding animal, amputation is certainly no more serious than a severe quittor or pricked foot. R. S. HUIDEKOPER, Veterinarian.

III.—INFLUENZA, CATARRHAL FEVER, &C.—As some, to me, interesting and strange complications have arisen in my practice from the above diseases, and may also prove of interest to readers of the JOURNAL, I send the following account: "On Feb. 28, 1887, I was called to treat a half-bred stallion, suffering from what they called "Pink Eye." On examination I found him with well marked symptoms of sore throat, eyes swollen, with a slight discharge of pus from the eyes and nostrils. He made a good recovery. In a few days, another stallion, one of the fashionable Daniel Lambert stock, developed same symptoms, but in about a week, to all appearances, regained his usual health. They were in a training stable, along with twelve other horses, and in a short time, every horse in the stable was affected in the same way; they all recovered except one, a valuable grey trotting mare, which died. The stallions were removed to their own stables, where I saw them occasionally. On the first of May they entered the stud, feeling and looking, so owner said, better than usual, at beginning of season. They were used to some forty mares, and *in every instance*, the mares that were covered by them, were attacked by the same disease from which the stallions suffered in February. The owner was obliged to withdraw the stallions, as it raised quite a disturbance among owners of the mares, some of whose young colts at their sides contracted the disease and died from it. These cases have puzzled me a good deal, as I have had considerable experience of above diseases in my practice, and never met with any case before, where the stallion would communicate it to mares. In 1874 I treated a large number of horses in Canada, among them a number of stallions, but never heard of them communicating it during the season ensuing. I trust the above may be of sufficient interest to the JOURNAL'S readers to induce them to communicate anything unusual in regard to above, that may have arisen in their practice.

R. LOVELL, V. S., Potsdam, N. Y."

